# Protein functional analysis data in support of comparative proteomics of the pathogenic black yeast *Exophiala dermatitidis* under different temperature conditions

**DOI:** 10.1016/j.dib.2015.08.008

**Published:** 2015-09-02

**Authors:** Donatella Tesei, Gorji Marzban, Martina Marchetti-Deschmann, Hakim Tafer, Elsa Arcalis, Katja Sterflinger

**Affiliations:** a VIBT Extremophile Center, Department of Biotechnology, University of Natural Resources and Life Sciences, Muthgasse 18, 1190 Vienna, Austria; b Plant Biotechnology Unit, Department of Biotechnology, University of Natural Resources and Life Sciences, Muthgasse 18, 1190 Vienna, Austria; c Institute of Chemical Technologies and Analytics, Vienna University of Technology, Getreidemarkt 9/164-IAC, 1060 Vienna, Austria; d Institute for Applied Genetics and Cell Biology, Department of Biotechnology, University of Natural Resources and Life Sciences, Muthgasse 18, 1190 Vienna, Austria

## Abstract

In the current study a comparative proteomic approach was used to investigate the response of the human pathogen black yeast *Exophiala dermatitidis* toward temperature treatment. Protein functional analysis – based on cellular process GO terms – was performed on the 32 temperature-responsive identified proteins. The bioinformatics analyses and data presented here provided novel insights into the cellular pathways at the base of the fungus temperature tolerance. A detailed analysis and interpretation of the data can be found in “Proteome of tolerance fine-tuning in the human pathogen black yeast *Exophiala dermatitidis*” by Tesei et al. (2015) [1].

## Specifications Table

**Table:** 

Subject area	Biology, Biotechnology
More specific subject area	Proteomics, Bioinformatics
Type of data	Overrepresented cellular process GO terms for the temperature-responsive proteins, predicted protein patterns and sub-cellular localization (excel tables)
How data was acquired	Bioinformatic analyses. Tools: Gostats, KOBAS, REVIGO, YLoc+
Data format	Raw and analyzed
Experimental factors	Thermo-tolerant black fungus and human pathogen *E. dermatitidis;* temperature treatments; bioinformatic analyses of the identified proteins
Experimental features	E. dermatitidis was exposed to temperature treatment – i.e. 45 and 1 °C - for 1 h and 1 week. After protein separation using DIGE and the identification of the differentially abundant proteins, bioinformatic analyses were performed to investigate the protein biological functions, pathways and sub-cellular localization.
Data source location	University of Natural Resources and Life sciences, Vienna, Austria
Data accessibility	Data are with this article and are related to [1]

## Value of the data

• The effect of temperature on *E. dermatitidis* proteome is investigated.
• Bioinformatic analyses are performed on the 32 identified temperature-modulated proteins.
• This approach allows to predict biological pathways, protein functions and sub-cellular localizations on the base of GO terms and protein sequences.
• The bioinformatics tools applied in the present study help generating, interpreting and validating biological information to be used for comparative proteomics studies
• The functional analysis of the temperature modulated proteins provides a better insight into the cellular pathways at the base of the fungus temperature tolerance
• The data are useful for comparing purpose when addressing the influence of diverse stress factors on the fungus protein expression

## 1 Data

In order to clarify the putative biological function of the identified temperature-modulated proteins and their involvement in particular cellular pathways, protein functional analysis was carried out on the base of cellular process GO terms.

Bioinformatic tools were applied in order to search for overrepresented cellular processes GO terms in the groups of protein showing increased or decreased abundance, to elucidate their putative biological functions. Lists of over-represented GO terms, where all proteins (indicated by the corresponding gene) enriched for a specific functional category are shown, were generated for each condition comparison (Table 1). Information about the GO Term ID, database and p-value are also available along with the hyperlink to the AmiGO2 application, where further details about each GO term are available. A similar list was created after categorization of the semantically related terms (Table 1).

On the base of the differentially abundant proteins the prediction of cellular pathways was also carried out. The obtained data, including information about significantly enriched GO pathways, pathway ID, database, number and list of genes regulated in the pathway, can be found in Table 2. Links to the respective annotated graphical pathway representations are additionally available.

Finally, the sub-cellular localization of the proteins identified by mass spectrometry was also performed. The sub-cellular location, probability and confidence of the prediction for each of the proteins are shown in Table 3.

## 2 Experimental design, materials and methods

The effects of different temperature conditions on the *E. dermatitidis* protein expression patterns have been analyzed by using a gel-based approach and by identifying temperature responsive proteins. Culture conditions and temperature treatments were performed as described in the Journal of Proteomics paper [1]. Briefly, after growing for 7 weeks at its temperature optimum (i.e. 37 °C), the strain was exposed to 1 °C and 45 °C both for 1 h and for 1 week. A number of 4 biological replicates – different petri dishes – were used for each experimental condition. 2D-DIGE and nLC-ESI-MS/MS were carried out to detect and identify proteins whose abundance changed by temperature treatment. Bioinformatic tools were applied in order to clarify the biological function of the 32 identified proteins and to predict their subcellular localizations and the pathways they are involved in.

## 3 GO terms

The FASTA sequence of each of the identified proteins was inputted into the UniProtKB database (http://www.uniprot.org/blast) in order to detect the respective Gene Ontology (GO) terms and annotation [2], [3]. In the case terms were not assigned to a protein, the most closely related protein sequence from a different organism, whose GO terms were available, was used. In the case GO terms were not accessible even for homologous proteins, the protein sequence was submitted as query to InterProScan 5 (http://www.ebi.ac.uk/Tools/pfa/iprscan5/) to scan for matches against the InterPro collection of protein signature databases using applications as PANTHER v9.0 (http://www.pantherdb.org) or SUPERFAMILY v1.75 (http://supfam.cs.bris.ac.uk/SUPERFAMILY/index.html). In the latter case, only terms with FDR <0.001 were selected.

## 4 Protein functional characterization

In order to elucidate the putative biological functions of the identified proteins, GOstats [4] and KOBAS v2.0 (http://kobas.cbi.pku.edu.cn) were used to search for overrepresented cellular processes GO terms in the group of increased and decreased proteins. GO terms with an uncorrected *p*-value <0.05 were considered significantly enriched. The complete list of over-represented cellular process GO terms per condition comparison is shown in Table 1 (raw data)

The lists of GO terms, were thereafter summarized with REVIGO (http://revigo.irg.hr) [5] by clustering semantically close GO terms (Table 1; summarized data) and shown in pie charts. The threshold for categorization was set to 0.5. In each pie chart, semantically close terms are clustered into categories of cellular processes, being each category represented by a different color (Fig. 6) in Tesei et al. [1].

## 5 Identification of most affected pathways

KOBAS was also applied to the prediction of enriched pathways – in the KEGG, BioCyc and Reactome database [6], [7], [8] – on the base of the set of up- and down-regulated genes. The genes were annotated with putative pathways by comparing them with genes with known annotation from *Saccaromyces cerevisiae* S288c. A pathway was considered significantly enriched when its uncorrected *p*-value was smaller than 0.05 [9]. A complete list of significantly enriched GO pathways and links to the respective annotated graphical representations are shown in Table 2. In each pathway the significantly regulated proteins are highlighted in blue and red according to decrease and increase in abundance, respectively. Green is used for the rest of the genes characterizing the pathway.

## 6 Protein sub-cellular localization

Information about the sub-cellular localization of the differentially abundant proteins was gained by using the YLoc prediction system (www.multiloc.org/YLoc) based on the YLoc+ model for fungal proteins [10]. The prediction was carried out according to the biological properties of each protein and performed into 10 different locations – also searching for multiple locations – taking into account GO terms transferred from close homologous proteins. Protein localizations for each condition comparison are shown in Table 6 [1]. The complete list of all identified proteins and respective sub-cellular localization is presented in Table 3.
