# Supplementary material for: Protein functional analysis data in support of comparative proteomics of the pathogenic black yeast Exophiala dermatitidis under different temperature conditions
Source: Data Brief. 2015 Sep 2;5:372–5. doi: 10.1016/j.dib.2015.08.008 (PMC4773397; doi:10.1016/j.dib.2015.08.008)
Supplement: Supplementary file 2 — Supplementary Material [file mmc2.zip › SupplementaryTable_3.docx]

**Supplementary Table 3**_ Sub-cellular localization of all identified proteins, as predicted by YLoc.

| **NCBI accession number** | **ORF** | **Protein name** | **Predicted Location^a^** | **Probability^b^**  **(%)** | **Confidence^c^** |
| --- | --- | --- | --- | --- | --- |
| gi\|378725637 | HMPREF1120_00315 | 14-3-3 family protein | cytoplasm | 91.34 | small (0.29) |
| gi\|378725903 | HMPREF1120_00576 | enolase | cytoplasm | 99.29 | very strong (0.96) |
| gi\|378726433 | HMPREF1120_01097 | 7-alpha-hydroxysteroid dehydrogenase | mitochondrion and cytoplasm | 45.68 | normal (0.35) |
| gi\|378726445 | HMPREF1120_01109 | aldehyde dehydrogenase | cytoplasm/peroxisome | 60.71 | normal (0.49) |
| gi\|378726610 | HMPREF1120_01270 | glyceraldehyde-3-phosphate dehydrogenase | cytoplasm | 91.20 | normal (0.49) |
| gi\|378726833 | HMPREF1120_01486 | hypoth. protein HMPREF1120_01486 | mitochondrion | 92.97 | normal (0.77) |
| gi\|378727822 | HMPREF1120_02451 | oxidoreductase, short-chain dehydrogenase/reductase | mitochondrion and cytoplasm | 40.48 | normal (0.46) |
| gi\|378727999 | HMPREF1120_02626 | hsp70-like protein | cytoplasm | 90.47 | strong (0.90) |
| gi\|378728030 | HMPREF1120_02657 | elongation factor 1-alpha | cytoplasm | 90.31 | strong (0.92) |
| gi\|378728179 | HMPREF1120_02805 | minor allergen Alt a 7 | extracellular space, cytoplasm, plasma membrane and nucleus | 20.76 | small (0.00) |
| gi\|378728843 | HMPREF1120_03444 | nucleoside diphosphate kinase | cytoplasm | 95.76 | strong (0.87) |
| gi\|378729152 | HMPREF1120_03741 | alcohol oxidase | peroxisome/cytoplasm | 85.63 | strong (0.84) |
| gi\|378729265 | HMPREF1120_03849 | ubiquinol-cytochrome c reductase sub. 7 | mitochondrion | 99.79 | very strong (1.00) |
| gi\|19073387 | HMPREF1120_04221 | HSP30 | cytoplasm/nucleus | 73.58 | strong (0.86) |
| gi\|378729668 | HMPREF1120_04224 | hypothetical protein HMPREF1120_04224 | cytoplasm and nucleus | 47.17 | small (0.19) |
| gi\|378729910 | HMPREF1120_04451 | haloalkanoic acid dehalogenase | cytoplasm/nucleus | 74.32 | strong (0.82) |
| gi\|378730051 | HMPREF1120_04591 | Glutathione S-transferase | cytoplasm/nucleus | 64.28 | normal (0.52) |
| gi\|378730392 | HMPREF1120_04915 | beta-lactamase | cytoplasm | 80.81 | normal (0.68) |
| gi\|378730528 | HMPREF1120_05043 | formate dehydrogenase | mitochondrion/cytoplasm | 67.52 | normal (0.52) |
| gi\|378732712 | HMPREF1120_07169 | proteosom component PUP2 | cytoplasm | 96.99 | strong (0.93) |
| gi\|378730713 | HMPREF1120_05220 | hypothetical protein HMPREF1120_05220 | cytoplasm/nucleus | 46.58 | normal (0.50) |
| gi\|378730838 | HMPREF1120_05340 | malate dehydrogenase | cytoplasm/mitochondrion | 61.81 | small (0.18) |
| gi\|378730878 | HMPREF1120_05378 | malate synthase, glyoxysomal | peroxisome | 98.33 | very strong (0.98) |
| gi\|378731564 | HMPREF1120_06041 | Phosphoenol pyruvate carboxykinase | cytoplasm/nucleus | 73.20 | strong (0.90) |
| gi\|378731617 | HMPREF1120_06094 | acetyl-coenzyme A synthetase | cytoplasm/nucleu/peroxisome | 57.92 | normal (0.70) |
| gi\|378731783 | HMPREF1120_06254 | mitochondrial protein-transporting ATPase | cytoplasm/nucleus | 85.12 | normal (0.47) |
| gi\|378731870 | HMPREF1120_06341 | hypoth. protein HMPREF1120_06341 | cytoplasm/peroxisome | 70.12 | normal (0.31) |
| gi\|378732911 | HMPREF1120_07361 | transketolase | cytoplasm, nucleus and peroxisome | 31.28 | small (0.19) |
| [gi\|378733644](file:///C:\Users\IAM\AppData\Local\Microsoft\Windows\Temporary%20Internet%20Files\pickinglist_09042014_45C1Wvs1C1W.xlsx#'45°C1Week vs 1C1W_UP'!Hit1) | HMPREF1120_08075 | hypoth. protein HMPREF1120_08075 | nucleus and cytoplasm | 49.62 | strong (0.86) |
| gi\|378734310 | HMPREF1120_08713 | tetrahydroxynaphthalene reductase | cytoplasm, nucleus and mitochondrion | 32.50 | small (0.21) |
| gi\|378734433 | HMPREF1120_08836 | Fe-Mn family superoxide dismutase | mitochondrion | 99.53 | very strong (0.99) |
| gi\|378734037 | HMPREF1120_08453 | transaldolase | cytoplasm/nucleus | 72.67 | normal (0.72) |
| **^a^ sub-cellular localization of the protein as predicted by YLoc+ according to the biological properties of the protein sequence** | | | | | |
| **^b^ probability of the sub-cellular location** | | | | | |
| **^c^ confidence that the prediction is reliable** | | | | | |
